# Molecular analysis of partial VP-2 gene amplified from rectal swab samples of diarrheic dogs in Pakistan confirms the circulation of canine parvovirus genetic variant CPV-2a and detects sequences of feline panleukopenia virus (FPV)

**DOI:** 10.1186/s12985-018-0958-y

**Published:** 2018-03-15

**Authors:** Nisar Ahmed, Adeel Riaz, Zahra Zubair, Muhammad Saqib, Sehrish Ijaz, Muhammad Shah Nawaz-Ul-Rehman, Ahmed Al-Qahtani, Muhammad Mubin

**Affiliations:** 1 Virology Lab, Center of Agricultural Biochemistry and Biotechnology (CABB), University of Agriculture, PO Box 38040, Jail road, Faisalabad, 38000 Pakistan; 2 Department of Infection and Immunity, Research Center, King Faisal Specialist Hospital and Research Center, Riyadh, Saudi Arabia; 3 Department of Microbiology and Immunology, Alfaisal University School of Medicine, Riyadh, Saudi Arabia; 4 Department of Clinical Medicine and Surgery, University of Agriculture, Faisalabad, Pakistan

**Keywords:** Canine parvovirus, Feline panleukopenia virus, Coinfection, VP2 gene and phylogenetic analysis

## Abstract

**Background:**

The infection in dogs due to canine parvovirus (CPV), is a highly contagious one with high mortality rate. The present study was undertaken for a detailed genetic analysis of partial VP2 gene i.e., 630 bp isolated from rectal swab samples of infected domestic and stray dogs from all areas of district Faisalabad. Monitoring of viruses is important, as continuous prevalence of viral infection might be associated with emergence of new virulent strains.

**Methods:**

In the present study, 40 rectal swab samples were collected from diarrheic dogs from different areas of district Faisalabad, Pakistan, in 2014–15 and screened for the presence of CPV by immunochromatography. Most of these dogs were stray dogs showing symptoms of diarrhea. Viral DNA was isolated and partial VP2 gene was amplified using gene specific primer pair Hfor/Hrev through PCR. Amplified fragments were cloned in pTZ57R/T (Fermentas) and completely sequenced. Sequences were analyzed and assembled by the Lasergene DNA analysis package (v8; DNAStar Inc., Madison, WI, USA).

**Results:**

The results with immunochromatography showed that 33/40 (82%) of dogs were positive for CPV. We were able to amplify a fragment of 630 bp from 25 samples. In 25 samples the sequences of CPV-2a were detected showing the amino acid substitution Ser297Ala and presence of amino acid (426-Asn) in partial VP2 protein. Interestingly the BLAST analysis showed the of feline panleukopenia virus (FPV) sequences in 3 samples which were already positive for new CPV-2a, with 99% sequence homology to other FPV sequences present in GenBank.

**Conclusions:**

Phylogenetic analysis showed clustering of partial CPV-VP-2 gene with viruses from China, India, Japan and Uruguay identifying a new variant, whereas the 3 FPV sequences showed immediate ancestral relationship with viruses from Portugal, South Africa and USA. Interesting observation was that CPV are clustering away from the commercial vaccine strains. In this work we provide a better understanding of CPV prevailing in Pakistan at molecular level. The detection of FPV could be a case of real co-infection or a case of dual presence, due to ingestion of contaminated food.

## Background

Canine parvovirus-2 (CPV-2) is a single stranded (ss) DNA virus that is the most dreadful enteropathogen causing myocarditis and acute hemorrhagic gastroenteritis in dogs and young pups while Feline panleukopenia is a disease of cats caused by a highly contagious virus i.e., feline panleukopenia virus (FPV) [1].

CPV belongs to family *Parvoviridae* within subfamily *Parvovirinae* under the genus *Protoparvovirus*, as a viral variant of *Carnivore protoparvovirus* 1 species along with other variant like FPV, mink enteritis virus (MEV) and raccoon parvovirus (RaPV) [2]. After CPV-2 emergence occurred, its further antigenic variants arose quickly in the field, CPV-2a (showing Met87Leu, Asn375Asp, Ile101Thr and Val555Ile amino acid variations) and CPV-2b (VP2 protein showing residue 426 changed from Asn426Asp and then from Asp426Glu in the CPV-2b and CPV-2c antigenic variant strains, respectively. However, as the strains CPV-2b and CPV-2c differ from CPV-2a by only one amino acid position (VP2 residue 426), they are not considered as distinct subtypes but only dominant variants of CPV- 2a [3, 4]. Antigenic variants, CPV-2a and CPV-2b divergence occurred from the CPV-2 strain due to changes in 5–6 amino acids in the antigenically important VP2 capsid region and in regaining the ability to infect feline cells in vitro and cats in-vivo again, while providing better adaptation to the canine host. CPV and FPV are two closely related viruses, causing disease in respective hosts, but new variants of CPVs have acquired the feline host range allowing them to infect both cats and dogs [5, 6], whereas the original CPV-2 does not replicate in cats. There are many studies showing that parvoviruses have a considerable degree of variability and that several viral variants can be detected simultaneously in an infected animal i.e., co-infection [1, 7–10]. Amplification of partial VP2 gene and subsequent sequencing of the PCR products, where many important informative amino acids reside, would give definitive antigenic and genetic difference between the original CPV-2, its variants, and the vaccine strains [11]. As mutation and recombination in VP2 gene influence the host range and affinity of receptor binding [12], it becomes important for researchers to sequence VP2 encoding gene for better and precise identification of emerging strains, their relationship with vaccine strains, and also to understand the evolutionary pattern of CPV strains in the field. For the purpose of identification of CPV, there are quite a few published papers where researchers have used partial VP2 gene sequence to report CPV [13, 14].

There is a recent study involving partial VP2 gene and amino acid analysis, where phylogenetic analysis had shown the circulation of heterogeneous CPV-2a strains in dog population in Pakistan. These strains shared high level of identity with Chinese strains [14]. The present study was undertaken for more detailed genetic analysis of partial VP2 gene sequences obtained from dogs and for possible co-infection of different parvoviruses, covering all areas of district Faisalabad. In this study we have reported phylogenetic and sequence analysis of two viruses i.e., CPV and FPV detected in dogs. The presence of a new CPV-2a variant was identified. The main objective of the study was to detect and genetically characterize prevalent parvoviruses circulating in Faisalabad, Pakistan.

## Methods

### Clinical samples

A total of 40 fecal samples/rectal swabs were collected from suspected dogs i.e., domestic as well as stray dogs from Veterinary Teaching Hospital, Department of Clinic Medicine and Surgery, University of Agriculture Faisalabad Pakistan and Civil Veterinary Hospital Faisalabad regardless of their vaccination status, as it was not available. In Pakistan more than 90% dogs are stray dogs with no vaccination what so ever. The samples were collected from dogs from different areas of Faisalabad district over the period of two years, (2014 and 2015). Five fecal samples/rectal swabs were collected from healthy dogs as negative control. The collected samples were emulsified in 0.1 M PBS and centrifuged at 3000 g for 10 min at 4C. The supernatant was used for further analysis.

### Qualitative detection of CPV antigens in feces of dogs

All the collected samples were checked for CPV Ag with the help of FASTest PARVO Strip (https://jfa.no/produkt/fastest-parvo-strip-2stk/), according to the instructions of manufacturer).

### DNA extraction and PCR amplification of partial VP2 gene

Viral DNA from fecal samples of infected and healthy dogs was extracted as described by [15]. To reduce the residual effect of DNA polymerase activity inhibitors the supernatants were diluted to a ratio of 1:10 using distilled water. A single step polymerase chain reaction (PCR) was performed to amplify viral DNA from clinical specimens. Gene specific primer pair Hfor/Hrev [16] were used to amplify 630 bp fragment of the capsid protein gene (VP2). The PCR-amplification of partial VP2 gene was carried out using 100 ng template DNA, in PCR mix as described earlier [16]. The PCR amplified products were run on 1.0% agarose gel containing ethidium bromide in Tris acetate EDTA (TAE) buffer and then visualized under UV transilluminator (Syngene, UK).

### Sequence and phylogenetic analysis

PCR amplified fragments were cloned in pTZ57R/T vector (Fermentas) and completely sequenced by chain termination method of sequencing on automated sequencer Applied Biosystem 3100. Sequences were analyzed and assembled by the Lasergene DNA analysis package (v8; DNA-STAR Inc., Madison, WI, USA). Phylogenetic trees were generated, first by aligning the molecules using CLUSTAL-W, followed by Maximum likelihood method of phylogenetic tree construction with 1000 bootstrap replications in MEGA7 program [17]. MegAlign program in DNA STAR was used to produce pairwise comparisons for amino acid sequence similarities. The accession numbers for CPV and FPV isolated in this study are provided in Tables 1 and 2. The other viral sequences used for amino acid sequence comparisons and phylogenetic analyses were downloaded from GenBank. The branch lengths were measured in terms of the number of substitutions per site.

**Table 1:** Details of samples sequenced, place and location, year of collection, gene bank accessions and mutations at various amino acid residues on partial VP2 protein of Canine parvovirus detected from dogs. The positions of mutations is based on CPV-2 GenBank accession number M38245, AY742953

S. No	Sample No/ Year of collection	Gene bank Accession numbers	Place of collection	CPV genome nucleotide/amino acid positions and mutations				CPV type
				3675/297 (T to G)	3756–3757/324 (TA to AT)	4062–4064/426 (AAT to GAT/GAA)	4105–4106/440 (AC to GC)	
1	01CPV2a15/2014	MF182906	Faisalabad	G	AT	GAT	GC	New2a (Ser297 → Ala)
2	04CPV2a15/2014	MF182907	Faisalabad	G	AT	GAT	GC	New2a (Ser297 → Ala)
3	05CPV2a15/2014	MF182908	Faisalabad	G	AT	GAT	GC	New2a (Ser297 → Ala)
4	06CPV2a15/2014	MF182909	Faisalabad	G	AT	GAT	GC	New2a (Ser297 → Ala)
5	07CPV2a15/2014	MF182910	Faisalabad	G	AT	GAT	GC	New2a (Ser297 → Ala)
6	09CPV2a15/2014	MF182911	Faisalabad	G	AT	GAT	GC	New2a (Ser297 → Ala)
7	13CPV2a15/2014	MF182912	Faisalabad	G	AT	GAT	GC	New2a (Ser297 → Ala)
8	14CPV2a15/2014	MF182913	Faisalabad	G	AT	GAT	GC	New2a (Ser297 → Ala)
9	15CPV2a15/2014	MF182914	Faisalabad	G	AT	GAT	AC	New2a (Ser297 → Ala)
10	16CPV2a15/2015	MF182915	Faisalabad	G	AT	GAT	GC	New2a (Ser297 → Ala)
11	17CPV2a15/2015	MF182916	Faisalabad	G	AT	GAT	GC	New2a (Ser297 → Ala)
12	18CPV2a15/2015	MF182917	Faisalabad	G	AT	GAT	GC	New2a (Ser297 → Ala)
13	19CPV2a15/2015	MF182918	Faisalabad	G	AT	GAT	GC	New2a (Ser297 → Ala)
14	20CPV2a15/2015	MF182919	Faisalabad	G	AT	GAT	AC	New2a (Ser297 → Ala)
15	21CPV2a15/2015	MF182920	Faisalabad	G	AT	GAT	GC	New2a (Ser297 → Ala)
16	23CPV2a15/2015	MF182921	Faisalabad	G	AT	GAT	AC	New2a (Ser297 → Ala)
17	24CPV2a15/2015	MF182922	Faisalabad	G	AT	GAT	GC	New2a (Ser297 → Ala)
18	25CPV2a15/2015	MF182923	Faisalabad	G	AT	GAT	GC	New2a (Ser297 → Ala)
19	26CPV2a15/2015	MF182924	Faisalabad	G	AT	GAT	GC	New2a (Ser297 → Ala)
20	28CPV2a15/2015	MF182925	Faisalabad	G	AT	GAT	GC	New2a (Ser297 → Ala)
21	32CPV2a15/2015	MF182926	Faisalabad	G	AT	GAT	GC	New2a (Ser297 → Ala)
22	33CPV2a15/2015	MF182927	Faisalabad	G	AT	GAT	GC	New2a (Ser297 → Ala)
23	34CPV2a15/2015	MF182928	Faisalabad	G	AT	GAT	GC	New2a (Ser297 → Ala)
24	38CPV2a15/2015	MF182929	Faisalabad	G	AT	GAT	AC	New2a (Ser297 → Ala)
25	39CPV2a15/2015	MF182930	Faisalabad	G	AT	GAT	AC	New2a (Ser297 → Ala)

**Table 2:** Details of samples sequenced, place and location, year of collection, gene bank accessions and mutations at position of amino acid residues on partial VP2 protein of Canine parvovirus and FPV detected from dogs

S. No	Sample No/ Year of collection	Gene bank Accession numbers	Place of collection	Amino acid positions					Serotype
				297	300	305	375	426	
1	10FPV15/2014	MF182903	Faisalabad	Ala	Gly	Tyr	Asp	Asn	FPV
2	11FPV15/2014	MF182904	Faisalabad	Ala	Gly	Tyr	Asp	Asn	FPV
3	13FPV15/2014	MF182905	Faisalabad	Ala	Gly	Tyr	Asp	Asn	FPV
4	FPV-314	D78585		Ala	Gly	Tyr	Asp	Asn	FPV
5	07CPV2a15/2014	MF182910	Faisalabad	Ser	Gly	Tyr	Asp	Asn	New2a (Ser297 → Ala)
6	09CPV2a15/2014	MF182911	Faisalabad	Ser	Gly	Tyr	Asp	Asn	New2a (Ser297 → Ala)
7	13CPV2a15/2014	MF182912	Faisalabad	Ser	Gly	Tyr	Asp	Asn	New2a (Ser297 → Ala)
8	CPV-16	M24003		Ser	Gly	Tyr	Asp	Asn	New2a (Ser297 → Ala)

## Results and discussion

### CPV antigen detection and PCR amplification

From 40 CPV suspected cases, fecal samples were collected and with the help of rapid immunochromatographic technique, 33 were found positive (82%) while 7 were negative (17%), 5 samples taken as negative control were found negative when analyzed using immunochromatographic technique. Of the total 33 positive samples, 25 samples, 75% (25/33), were confirmed positive for CPV as a fragment of 630 bp was amplified in all 25 samples. Amplified product from all samples were successfully cloned in pTZ57R/T (Fermentas) vector and completely sequenced.

### Sequencing analysis of CPV from Pakistan

VP2 gene amplified from representative isolates (total of 25) selected from diverse geographical locations of district Faisalabad was partially sequenced. After sequencing BLAST analysis showed 25 sequences from 25 different samples as the CPV new 2a variant and 3 sequences from 3 different samples, as the FPV. Three samples in which FPV was identified were also infected with the new CPV-2a variant. Of the 25 samples in which VP2 was amplified 100% (25/25) confirmed sequences with CPV 2a new variant and in 12% (3/25) sequences of FPV were identified. The partial VP2 nucleotide sequences from this study were analyzed using DNASTAR software, revealing homologies of 99.5–99.9% and 99.9% within the new CPV-2a strain solates and between the FPV isolates, previously reported from Asiatic region, mainly China and Europe. Similarly low nucleotide sequence similarity between the reference CPV 2a strain (AY742953), FPV (M38246) and CPV new 2a, FPV strains isolated in this study was found. All new CPV-2a clones had a nucleotide substitution at position 3675 (T-G); 3685 (C-G) and an identical nucleotide (T) at position 4064 (Table 1), which are characteristics of nucleotide variations of new CPV-2a (CPV-2a with nucleotide variation T-G at position 3675 or CPV-2a with amino acid variation Ser297-Ala). Multiple amino acid sequence alignment was performed to find sequence homology of the prevalent strains with each other and with reference CPV 2a (M24003), and a new CPV-2a (AY742953) and FPV (M38246) strains. Based on the amino acid residue at the 426 position of the VP2 gene, the isolates were classified into CPV-2a type (426-Asn) (Table 1). Additional amino acid changes were also found at 324 and 440 positions of the VP2 gene (Table 1). All of the new CPV 2a isolates in the present study had amino acid substitution Ser297Ala in VP2 (Table 1), indicating that the isolates were either new CPV-2a or new CPV-2b. All the new CPV 2a isolates reported during 2014–2015 had amino acid Tyr324Ile substitution due to mutation of TAT to ATT of the VP2 gene (Table 1). Residue 324 is a part of an important antigenic epitope and has ability to alter viral biology; it is under positive selection pressure [13] and arising independently in different geographic locations. Sequence analysis showed that this mutant is predominant in Asian countries like Korea, China and India. Emergence of this new variant is attributed to possible importation of foreign strains or could be the evolution of the existing CPV-2a strain. Further investigations are required to distinguish between these two possibilities; else this particular viral variant may spread throughout the Pakistan in the coming years. The neighboring residue 323, is known for binding to canine transferrin receptor TfR [18] along with residue 93 and have influence on viral host range and tissue tropism. Mutation in residue 323 from Asn to Asp causes changes in interactions between residues of elaborated loops within the same VP2 molecule or the other three fold VP2 molecule and hence could jeopardize virus multiplication in canine cells [19]. Twenty out of twenty-five isolates had an additional amino acid change 440 replacing Thr with Ala and further two out of those 20 isolates showed amino acid substitution Asp434Gly. Residue 440 gains the importance antigenically as it is present in GH loop of the VP2 protein which makes the surface of the virus capsid [20]. Residue 440 is present at the top of the three fold spike, which is considered to be the main antigenic site of the virus and also counts as one of the few VP sites that undergo positive selection [21]. Three isolate were found as Feline panleukopenia virus (Table 2), which mostly infects cats and cause gastroenteritis, was detected in three infected canine fecal samples. Previously it was known that two of CPV specific sequence amino acid at position 93-Asn and 323-Asn if introduced in FPV will allow it to multiply in-vitro canine cell line. These two amino acids along with 103-Val allow CPV to efficiently multiply in canine cells [19], but in the present study residue 323 remain FPV specific (Asp) and the remaining two amino acids were not determined in present sequence analysis. In addition, among the five amino acids in Table 2, the only difference between FPV and CPV from Pakistan as well as other regions is at the amino acid residue 297 (Ala in FPV and Ser in CPV isolate). On the other side, a study have shown that in-vivo inoculation of the FPV in dogs, unexpectedly enabled the virus to replicate in thymus and bone marrow cells but not in the intestine or mesenteric lymph nodes [22]. But, a recombinant virus of CPV and FPV was shown to multiply in high titers in canine intestine and mesenteric lymph nodes [23]. Two non-synonymous mutations, found in the present study, resulting in amino acid change Thr390Ala in (FPV sequence MF182903), Asp434Gly in (New CPV-2a sequence MF182909, MF182911, MF182917), which were previously not been reported in any other country.

### Phylogenetic analysis

**Fig. 1:**
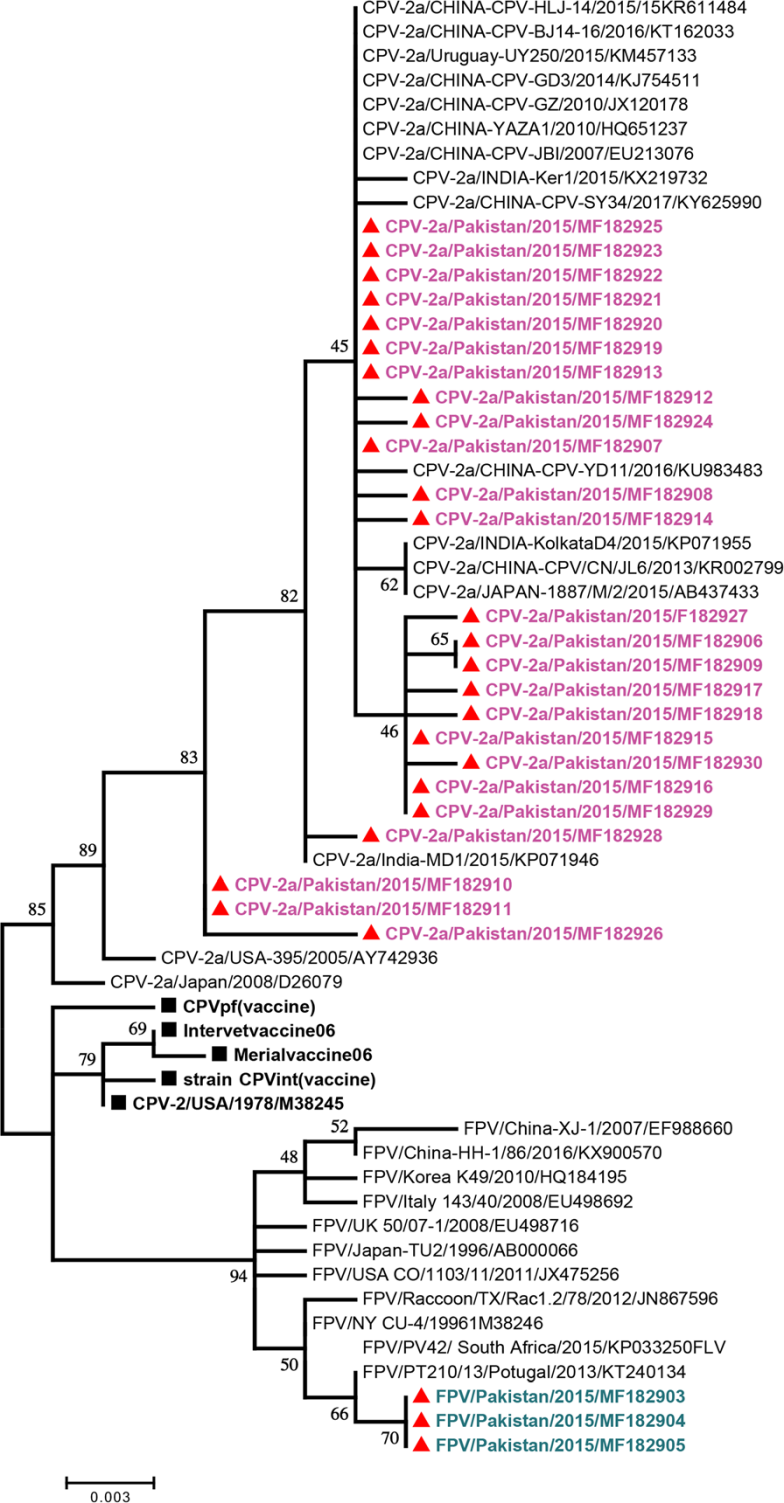
Maximum likelihood tree depicting phylogenetic relationship between CPV and FPV isolates from Pakistan and other geographical locations. Parvoviruses obtained from dogs sequenced in this study are shown with red triangles. Isolates with pink color show (CPV) and bold green colors show (FPV). The vaccine strains are shown with black squares. Bootstrap values are shown next to the branches in the phylogenetic tree

Maximum likelihood method was used to construct phylogenetic tree to determine the evolutionary relationship of our analyzed new CPV-2a strains with other strains of different geological areas along with vaccine strains. Three strains of FPV comprising 630 bp of partial VP2 gene from Pakistan along with other FPV strains of different geological areas were also included in the tree (Fig. 1). The parvoviruses sequenced in this study clustered in three different monophyletic clades in the phylogenetic tree at the antigenic region of VP2 gene though there is a very high sequence similarity among these sequences (Fig. 1). Total 25 isolates from 2014 to 15, could be typed as new CPV- 2a strains as shown in Fig. 1. These strains appeared to cluster with other CPV isolates from China, India, Japan, Thailand and Uruguay. These observations suggest that CPV-2a in Pakistan has maximum similarity with other strains of Asiatic origin. High nucleotides similarity between Indian, Chinese and Pakistani isolates of CPV-2a is an indication that this disease is endemic in the region. High nucleotides similarity with Indian isolates is not surprising, because there are similar environmental conditions in both the countries and it remained the same region for centuries. New CPV-2a isolates clustered with isolates of 2a or new 2a types from India, Uruguay and China. Thus the important observation, which is inferred from this phylogenetic analysis is that, the recent CPV isolates are clustering away from the commercial vaccine strains. Interestingly three FPV strains showed immediate ancestral relationship with viral strain reported from USA, Asia and Europe. The close clustering with these isolates suggests that FPV is highly conserved and should be addressed at global level. The mixed infection of FPV and CPV is surprising and interesting, as it has not been previously reported. The interesting observation is that the FPV alone was not detected in any of the samples. It was only detected in CPV positive samples. So, it might be possible that FPV was somehow supported by CPV. FPV alone might not be able to replicate in dogs. Further detailed experimentation is required to explain this phenomenon. There is another possibility to explain this observation. Dogs analyzed in this study were stray animals and due to coprophagous behavior in dogs it is very much likely that FPV detection was due to faeces ingestion and they are of no clinical relevance. However, further experiments are needed to support this observation.

## Conclusions

To date, there are no studies available on the clinical course and prognosis of cases presenting CPV-FPV dual infection. In this study CPV and FPV are detected in rectal swab samples of three dogs. We are not sure that whether presence of these two viruses together is just by chance or have some scientific relevance. If this is a true co-infection then we need to be very careful to study the epidemiology of these viruses. Analogous to what happens in the presence of multiple human infections [24], the clinical course of parvovirus infection could be altered and CPV could accelerate the progression of panleukopenia infection, since FPV represents a novel pathogen for dogs or vice versa. Alternatively, CPV-FPV co-infection could have a direct implication for virus persistence, host-viral interaction and pathogenesis [25]. As already discussed presence of these viruses together in rectal swab samples could be due to coprophagous behavior of dogs and FPV detection was due to faeces ingestion and they are of no clinical relevance. However, further sophisticated experiments are needed to support this observation.

## Abbreviations

BLAST: Basic local alignment search tool
CL: Color line
CPV: Canine parvovirus
FPV: Feline panleukopenia virus
MEV: Mink enteritis virus
PBS: Phosphate buffer saline
PCR: Polymerase chain reaction
RaPV: Raccoon parvovirus
TAE: Tris-acetate-EDTA
TL: Test line
